# 

**Published:** 1992-12

**Authors:** 


*New from Clinical Press*

### The Radiology of

### AIDS

In this book the wide range of radiological manifestations of AIDS are presented in the form of individual cases. The book will prove invaluable to any radiologist who wishes to familiarise himself with the varied radiological features of this disease.

By **A.K.Banerjee**

*80 Illustrations!*

This is a subject that every radiologist should know about.
This book should be in every radiology department library.
**Beautifully Illustrated**

**This book is now available at bookshops or may be ordered direct from Clinical Press Ltd, c/o Gazelle Book Services Ltd.,Falcon House,Queen Square, Lancaster LA1 1RN ,UK**

Paperbound. 140 pages. ISBN : 1 85457 025 0 *Only £ 15.00*

### Diagnostic Errors in Trauma Care

**And how to avoid them** ....Clear, concise and, above all, practical : *Only £12.50* **By H.R.Guly MBBS, MRCP**

Send this form to: Clinical Press Ltd, c/o Gazelle Book Services Ltd.,Falcon House,Queen Square, Lancaster LA1 1RN ,UK)

Please supply copy(ies) of the following titles:

-**Diagnostic Errors in Trauma Care**, Guly 1 85457 0 £12.50

-**The Radiology of Acquired Immune Deficiency**, Bannerjee 1 85457 025 0 £15.00

**Prepaid orders or credit-card orders will be supplied post free**

I enclose a cheque for ............................(payable to Gazelle Book Services)

OR

I authorise you to charge my Access/Barclaycard/American Express credit account

Card: ........................................................ Number: ....................................

Expiry date: ............................................. Signature: ..................................

OR

Please invoice me at list price plus £1.50 per copy postage and packing

Name (Please print)........................................................Address ..........................................

### MCQs for Part 1 FRCR

**Multiple Choice Questions for the First FRCR Examination**

**Jewell F.M., Pitcher E.M., Jones A.J.**

*Only £9.50* available directly from:

**Clinical Press Limited**
**Registered Office, Redland Green Farm**
**Redland Green, Redland, Bristol, BS6 7HF**

**Editorial Committee**
Chairman of Editorial Board, Professor John Farndon
Editor—Dr Paul Goddard
Assistant Editors—Mr M. G. Wilson, Dr Alison Round
Dr Julian Kabala, Dr James Catterall
Secretary—Mr Clive Ward
Member—Dr Kenneth Southgate

**Editorial Office**—*Redland Green Farm, Redland, Bristol BS6 7HF*

**Correspondents**
Dr Andrew Adam (Taunton)
Dr David Levine (Truro)
Dr Jack Davies (Bristol)
Professor Denis Pereira Gray (Exeter)
Dr Jose Jancar (Bristol)
Dr Michael Oliver (Barnstaple)
Mr Michael Reed (Gloucester)
Dr Michael Whitfied (Bristol)

**Associated Societies**
Bristol Medico-Chirurgical Society
Clinical Society of Bath
Severn Faculty of the Royal College of General Practioners
South Wales, South West and Wessex Rheumatology Club
South West of England and Wales Society of Dermatology
South West British Geriatric Society
South West Obstetricians' and Gynaecologists' Club
South West Orthopaedic Club
South West Paediatric Club
South West Radiologists' Association
South West Urologists' Club
South West Urological Oncology Group
South West Vascular Surgeons
Surgical Club of South West England
Tamar Faculty of the Royal College of General Practioners
Wessex Association of Clinical Pathologists
West Country Chest Physicians' Club
West Country Physicians' Club

Members of the above organisations receive the journal as part of their membership subscription.
Annual Subscription Rate £25.00 including surface postage.

**Published by**
Clinical Press,
*Redland Green Farm,*
*Redland Green, Redland, Bristol BS6 7HF.*
*Tel 0272 237709/420256*

**Typesetting and Printing by**
*H. E. ILES (Central Press) LTD.,*
*49/51 Downend Road,*
*Kingswood, Bristol BS15 1SF.*
*Tel 0272 614161 (4 lines)*

**THE WEST OF ENGLAND MEDICAL JOURNAL acknowledges with gratitude the help it receives from**

While the advice and information in this journal is believed to be true and accurate at the time of its going to press, neither the authors, the editors, nor the publisher can accept any legal responsibility for any errors or omissions that may be made. The publisher makes no warranty, express or implied, with respect to the material contained herein.

### WEST OF ENGLAND MEDICAL JOURNAL

1883—1992 Vols 1—107

### *Have you seen these articles?*

*Keep up to date in the use of MRI in your own speciality by reading Clinical MRI, the journal that shows you how to use magnetic resonance in a straightforward practical way.*

*Subscriptions £30 per annum*
*institutions £60*
*overseas (US) $75*

*From:*

*Redland Green Farm, Redland, Bristol, UK*

*BS6 7HF*

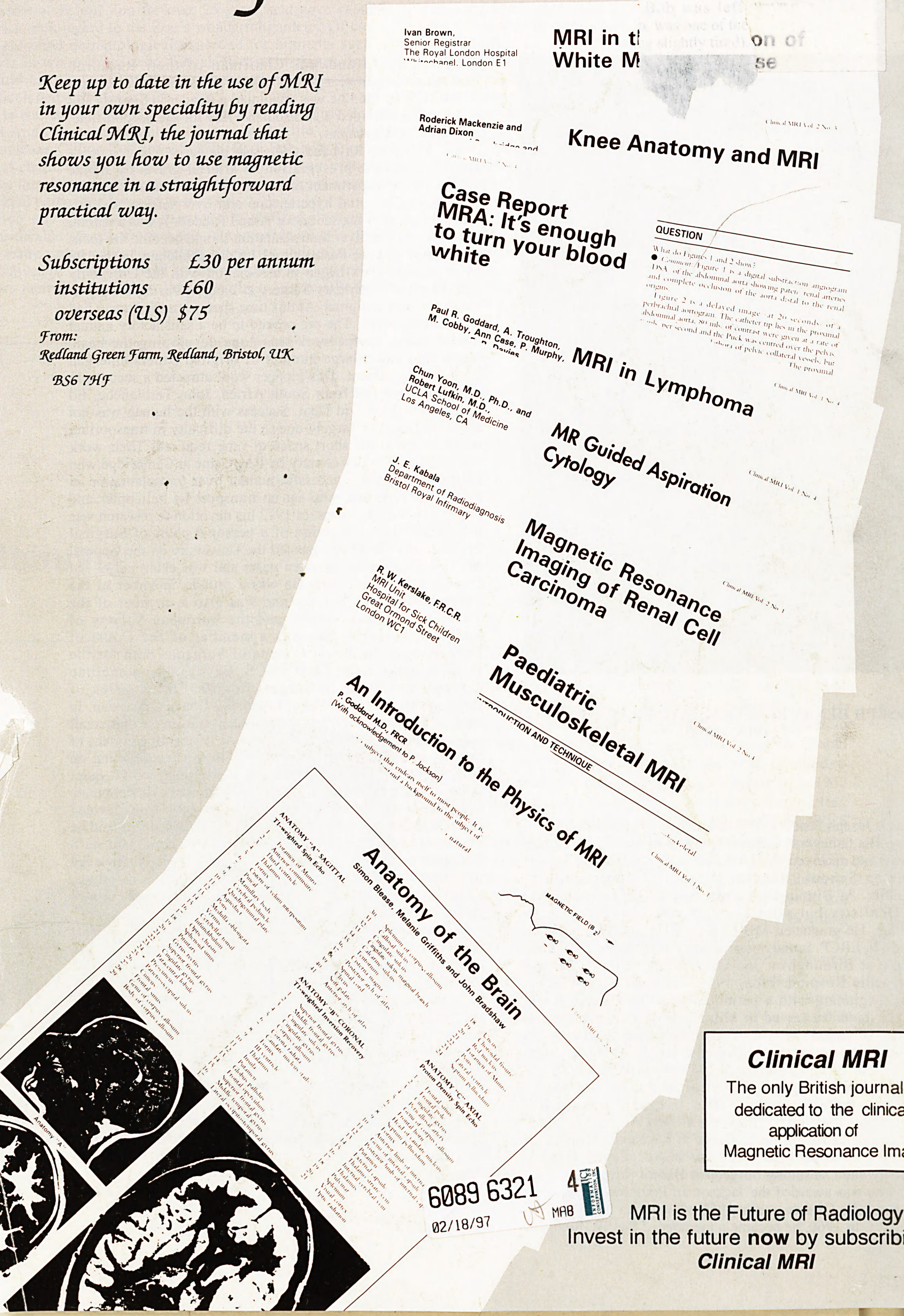

6089 6321 4

02/18/97 MAB

**Clinical MRI**

The only British journal dedicated to the clinical application of Magnetic Resonance Imaging

MRI is the Future of Radiology

Invest in the future **now** by subscribing t[illegible]

***Clinical MRI***

